# Polybrominated diphenyl ethers in relation to autism and developmental delay: a case-control study

**DOI:** 10.1186/1476-069X-10-1

**Published:** 2011-01-05

**Authors:** Irva Hertz-Picciotto, Åke Bergman, Britta Fängström, Melissa Rose, Paula Krakowiak, Isaac Pessah, Robin Hansen, Deborah H Bennett

**Affiliations:** 1 Department of Public Health Sciences, School of Medicine, University of California, MS1C, One Shields Ave., Davis, California, USA; 2 Department of Materials and Environmental Chemistry, Stockholm University SE-106 91 Stockholm, Sweden; 3 Department of Molecular Biosciences, School of Veterinary Medicine, University of California, Davis, California, USA; 4 Department of Pediatrics, School of Medicine, University of California, Davis, California, USA

## Abstract

**Background:**

Polybrominated diphenyl ethers (PBDEs) are flame retardants used widely and in increasing amounts in the U.S. over the last few decades. PBDEs and their metabolites cross the placenta and studies in rodents demonstrate neurodevelopmental toxicity from prenatal exposures. PBDE exposures occur both via breastfeeding and hand-to-mouth activities in small children.

**Methods:**

Participants were 100 children from the CHARGE (***CH***ildhood ***A***utism ***R***isk from ***G***enetics and the ***E***nvironment) Study, a case-control epidemiologic investigation of children with autism/autism spectrum disorder, with developmental delay and from the general population. Diagnoses of autism were confirmed by the Autism Diagnostic Observation Schedule and Autism Diagnostic Inventory-Revised, and of developmental delay using the Mullen's Scales of Early Learning and the Vineland Adaptive Behavior Scales. Typically developing controls were those with no evidence of delay, autism, or autism spectrum disorder. Eleven PBDE congeners were measured by gas chromatography/mass spectrometry from serum specimens collected after children were assessed. Logistic regression was used to evaluate the association between plasma PBDEs and autism.

**Results:**

Children with autism/autism spectrum disorder and developmental delay were similar to typically developing controls for all PBDE congeners, but levels were high for all three groups.

**Conclusions:**

Plasma samples collected post-diagnosis in this study may not represent early life exposures due to changes in diet and introduction of new household products containing PBDEs. Studies with direct measurements of prenatal or infant exposures are needed to assess the possible causal role for these compounds in autism spectrum disorders.

## Background

Few studies of autism have addressed environmental pollutants, and little research has been conducted in humans to assess neurodevelopmental toxicity of brominated flame retardants. Polybrominated diphenyl ethers (PBDEs) are flame retardants used widely in carpeting, foam furniture and car seats, textiles, plastic casings for television sets and computers, household appliances, and construction materials. The foam products have contained primarily penta-BDEs; plastic casings on small appliances and computers have been primarily octa-BDEs; and television sets, electrical wiring, and backings on draperies have generally been deca-BDEs [1]. Levels of PBDEs in human breast milk rose rapidly for several decades in Sweden but then declined, most probably in response to regulatory actions [2]. Concentrations reported in human blood are consistently higher in the U.S. than in Europe [3,4] and recent reports suggest that body burdens in California are among the highest worldwide [5-7]. Food, dust and air are the main routes of exposure [4,8].

PBDEs cross the placenta and are mobilized into breast milk during lactation [9,10]. A body of evidence has accumulated from experimental animal studies showing adverse neurodevelopmental consequences following prenatal and early life exposures [11-14]. These include hyperactivity and permanent alterations in spontaneous behaviors, deficits in learning and memory, and failure to habituate to novel stimuli, with effects seen at exposure levels comparable to those observed in humans [14]. In mice, exposures on postnatal days 3 and 10, but not postnatal day 19 altered motor behaviors, suggesting specific windows of vulnerability that translate to prenatal periods in humans [12].

Possible mechanisms relevant to neurodevelopmental toxicity include disruption of thyroid hormone or sex steroid homeostasis during the prenatal period [15-18], a period in which these systems play critical roles in fetal development, particularly in the formation of the external granule cell layer of the cerebellum [18]. PBDEs also show evidence of immunotoxic effects in rodent experiments [19]. Meanwhile, increasing research suggests neuroimmune pathways could contribute to autism [20,21]. In mechanistic studies, PBDEs also disregulate neuronal Ca^2+ ^signaling events [22,23], effects that appear to be magnified by hydroxylation of parent structure and that predict neurotoxic potential [22,24]. Several genes associated with autism risk are tightly regulated by Ca^2+ ^(e.g., neuroligin-3 [25]), or are themselves regulators of cellular Ca^2+ ^signals (e.g., Timothy Syndrome). Several congeners activate the pregnane X receptor, resulting in induction of cytochrome P450 enzymes [26].

Evidence of neurodevelopmental toxicity from prenatal exposures in animals, together with the abilities of PBDEs to disrupt endocrine systems and to interfere with immune development, provided the rationale for this pilot study of PBDEs and autism. This investigation builds on the Childhood Autism Risk from Genetics and the Environment (CHARGE) Study, which was initiated in 2002 as part of the Center for Children's Environmental Health at the University of California, Davis. Its overall goal, and that of the Center for Children's Environmental Health in general, is to identify factors that influence the incidence and severity of autism. We therefore compared levels of several PBDE congeners in cases to those in typically developing (TD) controls. Elsewhere, we reported that concentrations of PBDEs in this study are among the highest yet reported anywhere in the world [5].

## Methods

### Study design

This pilot project was designed to utilize the ongoing CHARGE Study, a comprehensive epidemiologic investigation of environmental factors in the etiology of autism. Launched in 2003, the CHARGE Study has enrolled well over 1000 children with the primary goal of identifying causes and contributing factors for autism, focusing especially on the chemical environment [27]. In addition, the CHARGE Study seeks to evaluate gene × environment interactions and to analyze phenotypic subtypes defined using behavioral or physiologic criteria, in order to determine whether environmental factors are more strongly related to specific subsets of autism cases.

The CHARGE Study uses a case-control design, enrolling families with an index child from one of three groups: children with autism, children with developmental delay but not autism, and children from the general population. All index children are between two and five years of age, reside in the study catchment area, were born in California, and are living with a biological parent who speaks either English or Spanish. The general population sample serves as a referent or control group.

### Recruitment and Data Collection

CHARGE Study recruitment of families with children in the autism/autism spectrum (AU/ASD) and developmental delay (DD) groups is largely through the California Department of Developmental Services (DDS) system, and through general publicity and other studies at the UC Davis M.I.N.D. (Medical Investigation of Neurodevelopmental Disorders) Institute. Population-based controls are selected by stratified random sampling from State birth files, with probabilities aimed at representing the overall distribution of autism cases with regard to age, gender, and broad geographic area of residence. Based on information in the birth record (for general population controls) or the DDS record (for the autism and DD groups), CHARGE Study staff search to locate the families and invite them to join the study.

All families are seen at the M.I.N.D. Institute where informed consent is administered and the child has a full psychometric evaluation and medical examination. We evaluate children with autism using the Autism Diagnostic Observation Schedules (ADOS) and interview the primary caregiver using the Autism Diagnostic Interview-revised (ADI-R); scores on these two instruments are used to confirm an autism or autism spectrum disorder diagnosis. A final diagnosis of autism (AU) is defined as meeting criteria on the communication, social, and repetitive behavior domains of the ADI-R with onset prior to 36 months and scoring at or above the communication and social interaction total cut off for autistic disorder on the ADOS module one or two. Children classified with autism spectrum disorders (ASD) are those who do not fully satisfy the criteria for autism but who do meet criteria on either the communication or the social interaction domain of the ADI-R prior to 36 months, are within two points of the cut-off on the other domain, and are above the social and communication total cutoff for ASD on the ADOS module one or two. Children with AU and ASD were combined into one group (AU/ASD) for statistical analysis of this pilot study. For children from the other two groups, the Social Communication Questionnaire (SCQ) is administered and if the score is above 14, the ADI-R and ADOS are administered to determine the final diagnosis for the CHARGE Study classification. Developmental delay is broadly defined as having a Mullen Scales of Early Learning (MSEL) Composite Score of less than 70, a Vineland Adaptive Behavior Scales (VABS) Composite of less than 70, and scoring above 14 on the SCQ. For this analysis, the delayed group also included children recruited from the DDS system who received services based on a diagnosis of developmental delay, had composite scores above the cutoffs and did not meet criteria for AU/ASD, as well as two children recruited from the general population with clear deficits on the MSEL (60 or lower). The typical development (TD) classification comprises children from the general population whose Composite scores on the MSEL *and *VABS were at or above 70. We categorized developmental regression status in children with autism using the ADI-R and the Early Development Questionnaire (EDQ). Regression versus early onset status was defined as loss of either language or social skills using ADI-R and EDQ questions [28].

Information on potential sociodemographic or environmental confounders was obtained from the birth certificate and from an extensive telephone interview with the primary caregiver. The interviewers were kept blind to the case status of the family as much as possible. Finally, biologic specimens were collected, including whole blood, which was drawn into yellow top acid citrate dextrose tubes and centrifuged. Plasma was stored at -80 degrees C for later analysis. Further details about recruitment and data collection in the CHARGE Study have been previously published [27]. All protocols were approved by the UC Davis School of Medicine and State of California Institutional Review Boards; data and specimens were collected only after informed consent was obtained.

### Pilot Sample

In the first funding period of the CHARGE Study (2001-2006), 799 children and their families participated. From these, we selected 100 children with blood samples collected from June 2003 to September 2005, in which sufficient volume of plasma was available. These were selected by stratified random sampling within strata of case groups: 50 children who entered the study with a diagnosis of autism, 25 with developmental delay and 25 from the general population. After clinical assessments and application of diagnostic criteria, the final study groups were: AU/ASD, n = 51, of whom 23 showed regression and the remaining 28 were categorized as early onset; developmentally delayed, n = 26; and typical development, n = 23.

### Laboratory methods for PBDE quantitation

Vials of serum (2-3 ml) were shipped to Sweden for chemical analysis of PBDEs. Samples were extracted and analyzed using gas chromatography with mass spectrometry. The extraction and cleanup procedure is described elsewhere [29]. Briefly, the surrogate standards (SS), BDE-77 (0.5 ng) and ^13^C-BDE-209 (1 ng) were added to 2-3 ml of plasma prior to extraction. The extracts were evaporated, and resolved in hexane. The neutral and phenolic substances were separated with potassium hydroxide (0.5 M in 50% EtOH) and hexane partitioning. The bulk of lipid in the neutral fraction was removed with concentrated sulfuric acid treatment. Additional cleanup was then preformed on a silica:sulfuric acid column (0.9 g). The silica gel column was always washed prior to sample application with the same solvent as the analytes were to be eluted with. The neutral fraction was fractionated on a column of activated silica gel (0.7 g). Most of the PCB congener and major conventional organochlorine pesticide interferences were eluted with hexane (3 ml) and the PBDEs were eluted with dichloromethane (8 ml). The solvent in the PBDE fraction was changed to hexane and reduced to 100 μl prior to GC/MS analysis. All samples were protected from daylight during handling and storage to prevent photochemical degradation of the brominated compounds to be analyzed.

Chemical standards were synthesized in-house for the individual congeners analyzed in this study, while BDE-209 was purchased from Fluka Chemie Buchs, Switzerland and ^13^C-labeled BDE-209 from Cambridge Isotope Laboratories, Andover, MA. All solvents were of pesticide quality. 2-Propanol from AnalaR (BDH laboratory supplies pool, England) and methyl tert-butyl ether (HPLC-grade; Rathburn, Walkerburn, Scotland) were glass-distilled prior to use. Silica gel (<0.063 mm) was purchased from Merck (Darmstadt, Germany) and activated at 300°C overnight, before it was used.

The PBDE analysis was performed by gas chromatography/mass spectrometry (GC/MS) utilising a Finnigan SSQ 700 instrument (ThermoFinnigan, Bremen, Germany) connected to a Varian 3400 gas chromatograph equipped with a CTC A200S autosampler. All instrumentation settings are reported elsewhere [2]. The PBDE congeners were analyzed with selected ion monitoring (SIM) by scanning for the negative bromide ion (isotopes m/z 79 and 81), formed by electron capture reactions at chemical ionization (ECNI) with methane (5.0, AGA, Stockholm, Sweden) as the electron thermalization buffer gas at 5.6 torr and a primary electron energy of 70 eV. For the BDE-209 analysis isotopic dilution in MS/ECNI was used by monitoring *m/z *484.2 and 486.2 for ^12^C-BDE-209 and *m/z *494.2 and 496.2 for ^13^C-BDE-209 [30]. All chromatographic data were collected, analyzed, and quantified using the proprietary ICIS2 software from Thermofinnigan.

Eleven PBDE congeners, BDE-28, BDE-47, BDE-66, BDE-85, BDE-99, BDE-100, BDE-153, BDE-183, BDE-197, BDE-207 and BDE-209, were analyzed with GC/MS (ECNI), as specified above, and quantified with the surrogate standards, BDE-77 and ^13^C-BDE-209. BDE-154 could not be quantified alone due to co-elution of the 2,2',4,4',5,5'-hexabromobiphenyl (BB-153) known to be present in human serum samples. Procedure solvent blank samples representing every seventh sample were analyzed in the same way as the serum extracts Limits of quantification (LOQs) were defined in direct relation to the amount of interference of PBDEs in the blank samples. The PBDEs in the samples had to be 3 times the concentration of the PBDEs in the blank to be considered for quantification. The LOQs were: for BDE-47, 0.03 ng, for BDE-207, 0.015 ng, for BDE-209, 0.01 ng; all other BDE congeners analyzed had LOQ values below 0.006 ng. In this study the LOQ values were set in direct relation to the amount of the PBDEs measured in the blank samples. The average blank sample amount has been subtracted from the results. Laboratory reference material was run in parallel to the analyzed samples.

We examined each BDE congener separately, and calculated several sums on a molar basis to take account variability in molecular weights. Because of low concentrations for BDE-183, only 64 samples yielded values above the limits of detection. For this reason, we calculated the sum of the seven measured lower brominated congeners from BDE-28 through BDE-153, and separately, the three measured octa- and deca-BDEs (BDE-197, -207, and -209).

### Lipids

Serum triglycerides and cholesterols were measured at the UC Davis clinical chemistry laboratory and total serum lipids were estimated [31]. Only 94 specimens have lipid values: two in the AU/ASD group, two in the DD group, and two in the TD group had insufficient sample volume for lipid determinations. Because PBDEs are lipophilic, concentrations are presented both on a wet weight basis (ng PBDEs/ml serum) and on a lipid basis (ng PBDEs/g lipids and pmol PBDEs/g lipids).

### Data Analysis

Laboratory data were received and merged with CHARGE Study files containing diagnostic and covariate information. All data were cleaned and verified, univariate distributions were reviewed for plausibility and consistency checks were performed. A log-transformation was applied to all individual BDE congeners and their sums due to the skewed distributions, as typically occurs with environmental contaminants.

To prepare for a multivariate analysis, we screened potential confounders. Our goal was to balance the need for a parsimonious model due to the small sample size in this pilot project with the importance of controlling actual confounding variables. Candidate confounders included mother's education and age at delivery, father's age, type of insurance payment for the delivery, number of computers in the household (as a measure of socioeconomic status), and child's age, sex, ethnicity, BMI, calendar time, and consumption of ocean fish. Those covariates showing associations with both PBDEs and case status were considered for inclusion in the multivariate analysis. Redundancy was assessed and a smaller set of variables was selected.

In the final analyses, we fit unconditional multivariate multinomial logistic regression models predicting the odds of autism or developmental delay relative to typical development as a function of PBDE concentrations with control for maternal education and calendar time. We calculated 90% confidence intervals, in recognition of the exploratory nature of this project. Analyses were weighted to reflect the socioeconomic distribution of the three target populations, i.e., children with autism, with developmental delay, or from the general population. Additional logistic regression models were fit to predict regressive AU vs. TD, and early onset AU vs. TD. As an exploratory analysis, we also subdivided the AU/ASD children by language development based on the criteria used for the choice of the ADOS module (i.e. nonverbal if module 1, verbal if module 2), to evaluate the association of PBDEs with these subtypes. ADOS Module 1 is intended for children who have limited use or understanding of words or for those who do not consistently use phrases of two or more words. Module 2 is administered to children who use some phrases and syntax consistently.

## Results

### Population Characteristics

Table 1 shows the characteristics of the study population. Three factors distinguished children with AU/ASD from typically developing children: maternal education, maternal age, and calendar date of blood draw. The mothers of children with AU/ASD were more likely than the mothers from either the DD or typically developing children to have had some college education (p = 0.03), while mothers of the DD children were the most likely to have had no education beyond high school. Additionally, mothers of AU/ASD, but not DD children, tended to be older (AU/ASD vs. TD, p = 0.09). Recruitment of autism cases began before recruitment from the other groups, resulting in significant differences in calendar time at blood draw. The deliveries of children with DD were somewhat less likely to be covered by private health insurance than deliveries of children in the other two groups, although the difference was not statistically significant.

**Table 1:** Characteristics of study sample (n = 94) by diagnostic status in the CHARGE Study, California, 2003 - 2005

	AU/ASD	DD N = 24	TD n = 21		
**Characteristic: Categorical variables**:	Weighted (unweighted) %†	Weighted (unweighted) %†	Weighted (unweighted) %†	AU/ASD *vs. *TD p-value†	DD *vs. *TD p-value†
Child's sex, male	92 (88)	68 (63)	86 (86)	0.81	0.19

Child's race/ethnicity					

White	30 (43)	18 (29)	32 (43)	0.94	0.56

Hispanic	45 (43)	60 (50)	48 (43)		

Other	25 (14)	22 (21)	20 (14)		

Mother's education					

≤ High school	12 (10)	44 (33)	36 (28)	0.03	0.10

Some college	60 (51)	45 (46)	26 (24)		

College or higher degree	28 (39)	11 (21)	38 (48)		

Computers in household					

None	13 (9)	37 (29)	24 (20)	0.61	0.35

One	52 (52)	24 (25)	47 (50)		

Two or more	35 (39)	39 (46)	29 (30)		

New car purchased in the first 2 years of child's life	21 (22)	24 (30)	10 (10)	0.29	0.24

Private insurance provider‡	72 (80)	50 (67)	74 (86)	0.88	0.17

Regional Center catchment area‡					

Alta, Far Northern, Redwood Coast	28 (31)	51 (54)	29 (29)	0.46	0.63

North Bay	20 (23)	5 (4)	11 (14)		

East Bay, San Andreas, Golden Gate	16 (10)	7 (8)	5 (10)		

Valley Mountain, Central Valley, Kern	18 (18)	17 (13)	34 (33)		

LA RCs*, Orange, San Diego, Tri-counties, Inland, San Gabriel/Pomona	18 (18)	20 (21)	21 (14)		

Child ate ocean fish (incl. tuna, caught) prior to age 2 yrs	27 (27)	39 (37)	39 (43)	0.37	0.99

Child ate freshwater fish (incl. caught) prior to age 2 yrs	8 (7)	7 (8)	8 (9)	0.99	0.85

Child's age, yrs	3.7 (0.1)	3.7 (0.2)	3.7 (0.1)	0.99	0.99

Child's weight, kg	17.1 (0.6)	15.4 (0.9)	16.9 (0.8)	0.85	0.23

Child's height, cm	99.5 (1.4)	96.4 (1.9)	99.2 (1.6)	0.90	0.26

Child's weight-to-height ratio, kg/m	16.9 (0.4)	15.8 (0.7)	16.9 (0.6)	0.92	0.25

Child's body mass index (BMI)	16.9 (0.2)	16.3 (0.6)	17.0 (0.5)	0.87	0.39

Length of breastfeeding, months	5.7 (0.6)	6.1 (1.7)	6.2 (1.9)	0.81	0.98

Mother's age, yr‡	30.4 (0.8)	28.3 (1.5)	27.7 (1.4)	0.10	0.79

Father's age, yr‡	32.5 (1.0)	30.7 (2.3)	30.4 (1.5)	0.25	0.92

Calendar years from first participant blood draw	1.0 (0.1)	1.2 (0.1)	1.4 (0.1)	0.006	0.34

†The weights were calculated to reflect the population targeted for recruitment. For children with AU/ASD or DD, this population was identified from the California DDS records for those who met our geographic and age criteria during the recruitment period. For children in the TD group, they were identified from State Birth Files, after restricting to those who met our geographic and age criteria during the recruitment period. The p-values were from comparisons of weighted proportions.

‡At the time of the child's birth.

*Los Angeles Regional Centers include Lanterman, Harbor, Westside, and Eastern, South Central, and North Los Angles

The children with autism were less likely to eat ocean fish (primarily tuna), but in this small sample, the differences were neither large nor statistically precise. With regard to mercury as a confounder, children with AU/ASD had similar levels of blood mercury. Moreover, blood Hg was not correlated with any of the PBDEs: Pearson correlation coefficients (with all variables log-transformed) ranged from -0.21 to 0.09. There were no significant differences across diagnostic groups with regard to father's age, or child's age, sex, race/ethnicity, region of California, height, weight, BMI, duration of breastfeeding, or consumption of freshwater fish.

### PBDE Concentrations

**Figure 1:**
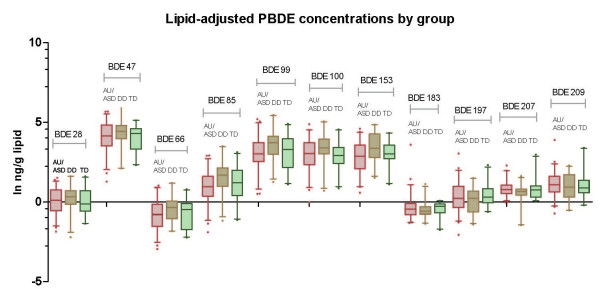
**Congener-specific PBDEs (ng/g lipids) by diagnostic group, children 24-60 months old, California, 2003-2005**. Individual congener distributions are shown on the log scale for PBDEs in ng/g lipids, measured in plasma taken from children participating in the CHARGE Study (n = 94). Differences among the three groups (AU/ASD, pink; DD, tan; TD, green), are minimal. All measurements were made in blood samples collected after the diagnosis.

**Figure 2:**
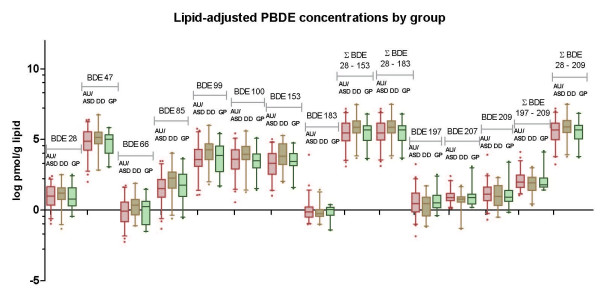
**Congener-specific PBDEs (molar basis: pmol/g lipid) *and selected sums *by diagnostic group, CHARGE Study children 24-60 months old, California, 2003-2005**. Congener distributions are shown on the log scale for PBDEs in pmol/g lipids, and sums of the lower brominated, higher brominated, or total measured PBDEs are also shown. Measurements were in plasma taken from children participating in the CHARGE Study (n = 94). Differences among the three groups (AU/ASD, pink; DD, tan; TD, green), are minimal. All measurements were made in blood samples collected after the diagnosis.

In unadjusted analyses, no individual PBDE congener (figures 1 and 2) or group of congeners (figure 2) differed comparing TD children with children having a confirmed AU/ASD diagnosis. A tendency towards higher concentrations of the low brominated congeners and lower concentrations of the more highly brominated PBDEs was observed among DD children (figure 1), but these were not significant. The distribution of PBDE congeners by diagnostic group is shown numerically in Additional file 1: Supplemental Table 1a (ng/g plasma), 1b (ng/g lipid), and 1c (pmol/g lipid).

Factors predictive of at least one PBDE congener were child's age, child's sex, maternal age, maternal education, duration of breastfeeding, and calendar time, and a few other socioeconomic variables. The only factors that could confound the PBDE × AU/ASD associations, based on their relationships to both PBDEs and child development, were maternal age and education, and for developmental delay, private insurance. Because of high correlations among maternal education, maternal age, and insurance status, we adjusted only for maternal education in the multivariate analyses. We also chose to include calendar year as a design variable that was strongly related to outcome. Additional adjustment for child's age, sex, or consumption of ocean fish, the type of health insurance used for the delivery hospitalization, as well as number of computers or other children in the household left results for PBDEs essentially unchanged.

After adjustment for maternal education and calendar time, children with autism still showed no significant differences in their serum PBDE concentrations relative to children with typical development, though in general, the levels were slightly lower (table 2). Comparing the 75^th ^vs. 25^th ^percentiles of PBDE levels, odds ratios for the AU/ASD group ranged from 0.49 (BDE #153, p = 0.12) and 0.50 (BDE #85, p = 0.11) to 0.87 (BDE #183, p = 0.66), and in all instances, the 90% confidence intervals easily overlapped the null value of 1.0. Odds ratios for DD vs. TD were above 1.0 for congeners with fewer than 8 bromines, and were below 1.0 for octa- through deca-BDEs, but again, all confidence intervals easily included the null. Only the odds ratio for BDE #197 (0.48) approached significance (p = 0.10).

**Table 2:** Multivariate models* predicting AU/ASD and DD the CHARGE Study, 2003-2005.

	Plasma Concentration**											
	ln (ng/g lipid)	ln (pmol/g lipid)	AU/ASD *vs. *TD					DD *vs. *TD				
**Predictor variable**	**(25**^**th**^**, 75**^**th**^**)**	**(25**^**th**^**, 75**^**th**^**)**	**β**	**SE**	**OR**†	**90% CI**	**p-value**	**β**	**SE**	**OR**†	**90% CI**	**p-value**

BDE 28	(-0.47, 0.70)	(0.43, 1.60)	-0.17	0.38	0.82	(0.39, 1.71)	0.66	0.34	0.49	1.48	(0.58, 3.82)	0.49
BDE 47	(3.56, 4.75)	(4.28, 5.47)	-0.24	0.32	0.75	(0.40, 1.42)	0.46	0.35	0.44	1.52	(0.65, 3.58)	0.42
BDE 66	(-1.41, -0.11)	(-0.69, 0.61)	-0.39	0.38	0.60	(0.27, 1.36)	0.30	0.48	0.42	1.86	(0.76, 4.59)	0.26
BDE 85	(0.53, 1.91)	(1.10, 2.48)	-0.50	0.31	0.50	(0.25, 1.02)	0.11	0.32	0.39	1.55	(0.64, 3.77)	0.42
BDE 99	(2.57, 3.90)	(3.14, 4.47)	-0.33	0.32	0.65	(0.32, 1.31)	0.31	0.46	0.38	1.84	(0.80, 4.25)	0.23
BDE 100	(2.61, 3.68)	(3.18, 4.25)	-0.32	0.31	0.71	(0.41, 1.23)	0.31	0.41	0.43	1.55	(0.72, 3.34)	0.34
BDE 153	(2.47, 3.61)	(2.91, 4.05)	-0.63	0.41	0.49	(0.23, 1.05)	0.12	0.61	0.48	1.99	(0.81, 4.91)	0.21
BDE 183	(-0.77, -0.18)	(-0.44, 0.15)	-0.25	0.56	0.87	(0.50, 1.49)	0.66	0.05	0.53	1.03	(0.62, 1.72)	0.92
Σ BDE 28 - 153	-	(5.08, 6.10)	-0.34	0.37	0.71	(0.38, 1.32)	0.36	0.51	0.46	1.69	(0.78, 3.65)	0.26
Σ BDE 28 - 183	-	(5.11, 6.10)	-0.34	0.38	0.71	(0.38, 1.33)	0.37	0.52	0.46	1.67	(0.78, 3.58)	0.26
BDE 197	(-0.35, 0.81)	(-0.13, 1.04)	-0.44	0.39	0.60	(0.28, 1.26)	0.25	-0.63	0.39	0.48	(0.23, 1.00)	0.10
BDE 207	(0.50, 1.00)	(0.63, 1.13)	-0.42	0.55	0.81	(0.51, 1.28)	0.45	-0.86	0.60	0.65	(0.40, 1.07)	0.15
BDE 209	(0.53, 1.58)	(0.57, 1.62)	-0.42	0.42	0.65	(0.31, 1.34)	0.32	-0.49	0.46	0.60	(0.27, 1.32)	0.29
Σ BDE 197 - 209	-	(1.61, 2.44)	-0.51	0.52	0.65	(0.32, 1.34)	0.33	-0.76	0.56	0.53	(0.25, 1.15)	0.18
Σ BDE 28 - 209	-	(5.15, 6.12)	-0.35	0.39	0.71	(0.38, 1.33)	0.37	0.51	0.47	1.64	(0.77, 3.50)	0.28
Mother's education‡												
≤ high school	-	-	-0.60	0.85	0.55	(0.13, 2.23)	0.48	1.50	0.87	4.49	(1.07, 18.90)	0.09
some college	-	-	1.10	0.73	3.01	(0.90, 10.01)	***0.13***	1.75	0.86	5.75	(1.40, 23.59)	0.04
Calendar time‡	-	-	-1.11	0.55	0.33	(0.13, 0.81)	***0.04***	-0.67	0.68	0.51	(0.17, 1.56)	0.32

*Each congener or subset of congeners was entered, log-transformed and lipid-adjusted, into a separate multinomial logistic regression model to predict AU/ASD or DD.

** Percentiles are presented as log-transformed values of concentrations in lipids

† Odds ratio for difference in PBDE levels comparing those at 75^th ^and 25^th ^percentiles

‡All models were fitted with multivariate adjustment for mother's education (reference group is college degree) and calendar time, and the results for these factors were similar across models. What we present in the table was a model with mother's education and calendar time without PBDE.

### Phenotypic Subsets of Autism

In further analyses, we separated the early onset from regressive cases of autism. The two subtypes were similar in their distributions of PBDEs, and in multivariate-adjusted analyses, neither group differed from TD controls for any PBDE or group of PBDEs (data not shown). Alternative analyses dividing the case group by language development and controlling for child's age, a strong predictor of verbal skills, yielded no evidence of differences in PBDEs as compared with TD controls.

## Discussion

As previously described, PBDEs are widespread in household products and have now moved into the food chain [5], although little research has addressed human health effects. The lack of association between children's circulating levels of PBDEs and autism case status does not preclude a role for PBDEs in autism etiology. A weakness in this pilot study was the examination of current levels of PBDEs as a proxy for exposures that preceded the neuropathologic changes leading to autism. In retrospective research such as the CHARGE Study and other case-control investigations, obtaining etiologically relevant exposure measurements is challenging. Half-lives of PBDEs vary. Higher brominated compounds appear to have the shortest half-lives, e.g., 15 days for #209, 39 days for #207, and 94 days for #183, based on a study involving occupational exposures [32] and therefore, correlations of internal measurements over a period of years would not be expected to be high. Half-lives of lower brominated congeners were estimated based on daily intake and total body burden as 1.8, 2.9, 1.6, and 6.5 years for BDE-47, -99, -100, and -153, respectively [33]. Nevertheless, even for those compounds that are retained, correlations over time can vary widely and will be a function primarily of the introduction of new sources, elimination of old sources, and changes in behaviors that influence extent of contact with those sources. The correlation between current and past exposure will be greatest when the sources are constant over time for an individual, or when external sources have been eliminated and only past exposures are present, with gradual ongoing excretion. Exposures were rising in the U.S. between the births and the time of data collection for these subjects, as a result of continued production of household furnishings and electronics with PBDEs, and increasing levels in the food supply, and therefore, it is unlikely participants' exposures remained steady [3]. We also note that the concentrations in this sample are, to our knowledge, higher than previously reported in any other population, even from the 2003-2004 NHANES sample [34], and are seven to ten times higher than those reported to predict lower cognitive scores in a sample of children from New York City [35].

During the prenatal period, the mother's exposures will be critical. Her exposures will be partially based on diet, and partially based on pathways related to the home environment, including inhalation of both chemicals in the gas phase and resuspended particulate matter, as well as dermal exposure and non-dietary ingestion of dust. During early infancy, exposures, particularly for lower brominated congeners [36] appear to be primarily through breast milk [8], again reflective of the mother's exposure, or infant formula, as well as some contribution from inhalation of air and dust, possibly from mattresses, upholstered furniture, and cushions. With the introduction of solid food, the diet becomes more varied, with new sources of PBDE exposures, even though some toddlers choose to eat a limited number of food items. As children start to interact more independently with their environment toward the second half of the first year, intake becomes increasingly influenced by what is present in the home environment, in large part due to increases in frequency of contact with surfaces, hand to mouth activity, and object to mouth activity [37,38]. Higher concentrations have been found in toddlers than in school-age children or adults, supporting a critical role for non-food ingestion during the early years [36]. All of these factors underscore the differences between exposures measured at 24 to 60 months of age versus those that occurred during a critical time period, which may very well be the prenatal period. The issue is further complicated when comparing children with developmental disorders to those with typical development: the former may have different timing or frequency in their hand to mouth activity, delays in explorative play, or a more limited diet due to feeding difficulties [39-41]. Similar to what others have reported, in our study sample, both food and non-food sources appeared to contribute to the children's body burdens [5].

Given the uncertainties outlined above regarding exposure and intake, particularly for infants and toddlers, and the dramatic changes between birth and age two-five years in diet and play behaviors, current PBDE concentrations (even when half-lives are long, e.g., 4 years) may not adequately estimate exposures in the prenatal or early postnatal period. House dust or banked specimens from adults (whose diet and behaviors change less than their children's) might provide better estimates of PBDE exposures during the critical time windows. Breakdown of the deca-BDE (209) may occur in house dust, but for other congeners, stability of levels in house dust may be an indication that it serves as a more valid repository of long-term exposures than blood plasma, at least in young children [42].

Although few risk factors have been established for autism, we evaluated more than ten potential confounders that had no appreciable effect on the coefficients relating PBDEs to diagnosis. Similar to the findings at an older age [43], during the first two years of life, children with autism were less likely to eat ocean fish (primarily tuna), but in this small sample, the differences were neither large nor statistically precise. As Hg was not associated with case status and showed low correlations with PBDEs, it could not have confounded the analyses of PBDEs and AU/ASD or developmental delay. Confounding by known risk factors or other sociodemographic characteristics seems unlikely to have been responsible for the null results of this study.

Another consideration in the interpretation of these null findings is that the levels of PBDEs may be sufficiently high that all children who are predisposed to develop autism have had exposures sufficient to surpass their individual thresholds. It has previously been noted [44] that when exposure is high and ubiquitous, no study will be able to detect the effect of that exposure; within such populations, diseases will instead appear to have only genetic influences. Despite the speculative nature of this proposition in the case of autism and PBDEs, the problem of widespread environmental chemical pollution poses a serious obstacle to identifying health effects. Even their interaction with genes will be elusive and research could appear to support a purely genetic etiology either when the exposure variability is low, or when levels are so high as to exceed the threshold in all susceptible individuals.

The lower concentration of BDE #197 in children with developmental delay is most likely a chance finding, given the small sample size in this pilot study and the large number of comparisons made. Children with developmental delay often have more, rather than less, hand-to-mouth activity, thus it is unclear whether behaviors could explain this particular association. For the lower brominated congeners, the more extreme values in some children from the DD group is interesting, and might reflect different behavioral patterns in children with cognitive and adaptive impairments.

Thus, although our current measurements of PBDEs do not predict risk of autism, this pilot project should not be construed as the last word on PBDEs and autism. On the one hand, our null findings could have been the consequence of substantial misclassification relative to exposure in the etiologically critical time windows: measurements in plasma samples collected at least a year and potentially as much as five years after the key events in neurodevelopment may have been poor approximations of the relevant prenatal or early postnatal exposures. On the other, the biological plausibility of a causal association between prenatal or early postnatal PBDE exposures and the development of autism is supported by numerous documented toxicologic mechanisms relevant to CNS development.

Further research can be directed towards several issues: a better understanding of sources and pathways of exposure for pregnant women and young children; design of methods to obtain more accurate measurements of exposure, especially to hydroxylated metabolites, during periods critical for autism etiology; analysis of larger sample sizes; and expansion of animal and in vitro models of neurodevelopmental toxicity to define the molecular mechanisms. Of potential relevance are Ca^2+ ^signaling pathways, endocrine disruption, or other cellular, intracellular and transport processes, and their regulation by genes that may have been associated with autism.

## Conclusions

Although this study found no association between children's circulating levels of PBDEs and autism case status, further work investigating a role for PBDEs in autism etiology is warranted for several reasons: exposures in the U.S. remain high; data from animals [11,14] and humans [35] have shown neurodevelopmental effects following prenatal exposures; and experimental research suggests several relevant mechanisms of toxicity [15,22-25]. The underlying assumption of the analyses presented here was that PBDEs in children 24-60 months could serve as a proxy for exposures that preceded the neuropathologic changes leading to autism, e.g., in prenatal or early postnatal life. Recent reports [5,8,45], indicate that diet may contribute substantially to children's PBDE concentrations. Given that major dietary changes occur during the first few years of life, and that maternal pregnancy food consumption may be quite dissimilar from children's intake in their third to fifth year of life, it seems probable that exposure was misclassified. Future research should seek other methods to obtain etiologically relevant measurements of PBDE exposures in case-control studies, and opportunities to assess these compounds in prospective investigations of ASD.

## Abbreviations

AU: autism; ASD: autism spectrum disorders; DD: developmental delay; TD: typical development; PBDE: polybrominated diphenyl ethers; BDE: brominated diphenyl ether; Ca^2+^: calcium ions; CHARGE: CHildhood Autism Risks from Genetics and the Environment; LOQ: limits of quantitation; MS/ECNI: mass spectrometry, electron capture negative ionization; GC/MS: gas chromatography, mass spectrometry; EtOH: ethanol; SCQ: Social Communication Questionnaire; EDQ: Early Developmental Questionnaire; ADOS: Autism Diagnostic Observation Schedule; ADI-R: Autism Diagnostic Inventory-Revised; MSEL: Mullen Scales of Early Development; VABS: Vineland Adaptive Behavior Scales; DDS: Department of Developomental Services;

## Competing interests

The authors declare that they have no competing interests.

## Authors' contributions

IHP was responsible for conception, acquisition of funding, and general supervision of the research group. AB directed the laboratory where PBDEs were assayed and supervised BF, who conducted the chemical determinations, and they also wrote parts of the methods section. MR and DB contributed to the introduction and discussion regarding sources of exposure, behavioral modifiers, and time trends. PK conducted all of the statistical analyses, wrote parts of the statistical methods section, and produced the tables and figures. INP wrote sections of the introduction and discussion relating to toxicity. RH directed the clinical assessments and application of diagnostic criteria for autism spectrum disorders, developmental delay, typical development, and autistic regression. All authors read and approved the final manuscript.

## Supplementary Material

Additional file 1 **Supplemental Tables**. The descriptive statistics of the distribution of PBDE congeners is shown by diagnostic group for three different metrics of these compounds (ng/g plasma; ng/g lipids; pmol/g lipids). Click here for file
