# Supplementary material for: Polybrominated diphenyl ethers in relation to autism and developmental delay: a case-control study
Source: Environ Health. 2011 Jan 5;10:1. doi: 10.1186/1476-069X-10-1 (PMC3029221; doi:10.1186/1476-069X-10-1)
Supplement: Additional file 1 — Supplemental Tables. The descriptive statistics of the distribution of PBDE congeners is shown by diagnostic group for three different metrics of these compounds (ng/g plasma; ng/g lipids; pmol/g lipids). [file 1476-069X-10-1-S1.DOC]

Supplemental Tables: The descriptive statistics of the distribution of PBDE congeners is shown by diagnostic group for three different metrics of these compounds (ng/g plasma; ng/g lipids; pmol/g lipids).

Supplemental Table 1a: Comparison of mean BDEs (*ng/g plasma*) across three groups*

|  | **AU/ASD (n=49)** | | |  | **DD (n=24)** | | |  | **TD (n=21)** | | |
| --- | --- | --- | --- | --- | --- | --- | --- | --- | --- | --- | --- |
| **PBDE congener** | **Mean(SD)** | **Med (25^th^,75^th^)** | **(5^th^,95^th^)** |  | **Mean(SD)** | **Med (25^th^,75^th^)** | **(5^th^,95^th^)** |  | **Mean (SD)** | **Med (25^th^,75^th^)** | **(5^th^,95^th^)** |
| BDE 28 | 0.01(0.01) | 0.01 (0.00,0.01) | (0.00,0.02) |  | 0.01(0.01) | 0.01 (0.01,0.01) | (0.00,0.02) |  | 0.01 (0.01) | 0.01 (0.00,0.01) | (0.00,0.02) |
| BDE 47 | 0.42(0.39) | 0.31 (0.17,0.57) | (0.06,1.05) |  | 0.59(0.54) | 0.39 (0.28,0.65) | (0.05,1.66) |  | 0.38 (0.23) | 0.43 (0.19,0.48) | (0.06,0.82) |
| BDE 66 | 0.00(0.00) | 0.00 (0.00,0.00) | (0.00,0.01) |  | 0.01(0.01) | 0.00 (0.00,0.01) | (0.00,0.01) |  | 0.00 (0.00) | 0.00 (0.00,0.01) | (0.00,0.01) |
| BDE 85 | 0.02(0.03) | 0.01 (0.01,0.02) | (0.00,0.11) |  | 0.04(0.04) | 0.03 (0.02,0.05) | (0.00,0.13) |  | 0.03 (0.03) | 0.02 (0.01,0.04) | (0.00,0.05) |
| BDE 99 | 0.19(0.24) | 0.11 (0.05,0.20) | (0.02,0.73) |  | 0.29(0.32) | 0.18 (0.12,0.34) | (0.04,0.85) |  | 0.18 (0.15) | 0.16 (0.07,0.24) | (0.02,0.36) |
| BDE 100 | 0.15(0.16) | 0.10 (0.06,0.18) | (0.02,0.43) |  | 0.22(0.20) | 0.16 (0.11,0.26) | (0.02,0.61) |  | 0.13 (0.10) | 0.11 (0.07,0.16) | (0.03,0.33) |
| BDE 153 | 0.13(0.12) | 0.08 (0.04,0.15) | (0.02,0.37) |  | 0.21(0.16) | 0.15 (0.09,0.28) | (0.04,0.50) |  | 0.14 (0.10) | 0.13 (0.08,0.16) | (0.03,0.35) |
| BDE 183 | 0.01(0.02) | 0.00 (0.00,0.00) | (0.00,0.01) |  | 0.00(0.00) | 0.00 (0.00,0.00) | (0.00,0.01) |  | 0.00 (0.00) | 0.00 (0.00,0.00) | (0.00,0.01) |
| BDE 197 | 0.01(0.02) | 0.01 (0.00,0.01) | (0.00,0.03) |  | 0.01(0.01) | 0.01 (0.00,0.01) | (0.00,0.02) |  | 0.01 (0.01) | 0.01 (0.01,0.01) | (0.00,0.03) |
| BDE 207 | 0.01(0.01) | 0.01 (0.01,0.01) | (0.01,0.03) |  | 0.01(0.01) | 0.01 (0.01,0.01) | (0.00,0.02) |  | 0.02 (0.02) | 0.01 (0.01,0.01) | (0.01,0.04) |
| BDE 209 | 0.02(0.04) | 0.01 (0.01,0.02) | (0.01,0.04) |  | 0.02(0.01) | 0.01 (0.01,0.03) | (0.01,0.04) |  | 0.03 (0.05) | 0.01 (0.01,0.02) | (0.00,0.13) |

*For some PBDEs, the rank ordering of the three groups based on the means differs from that based on the medians due to differences in the tails of the distributions.

Supplemental Table 1b: Comparison of mean BDEs (*ng/g lipid*) across three groups*

|  | **AU/ASD (n=49)** | | |  | **DD (n=24)** | | |  | **TD (n=21)** | | |
| --- | --- | --- | --- | --- | --- | --- | --- | --- | --- | --- | --- |
| **PBDE congener** | **Mean(SD)** | **Med (25^th^,75^th^)** | **(5^th^,95^th^)** |  | **Mean(SD)** | **Med (25^th^,75^th^)** | **(5^th^,95^th^)** |  | **Mean(SD)** | **Med (25^th^,75^th^)** | **(5^th^,95^th^)** |
| BDE 28 | 1.42(1.08) | 1.11 (0.61,2.13) | (0.25,3.35) |  | 1.75(1.33) | 1.38 (0.94, 1.98) | (0.37,4.86) |  | 1.38(1.15) | 0.88 (0.58, 1.87) | (0.30,2.67) |
| BDE 47 | 85.19(71.11) | 63.39 (34.36,117.18) | (9.70,222.98) |  | 113.42(105.21) | 82.19 (54.61, 119.69) | (8.61,382.93) |  | 73.38(49.22) | 73.62 (30.07, 98.16) | (14.88,165.25) |
| BDE 66 | 0.65(0.66) | 0.45 (0.23,0.87) | (0.09,2.44) |  | 0.94(0.84) | 0.70 (0.39, 1.00) | (0.16,3.07) |  | 0.67(0.53) | 0.62 (0.20, 0.80) | (0.13,1.48) |
| BDE 85 | 4.30(4.43) | 2.60 (1.54,4.75) | (0.38,12.75) |  | 7.60(8.07) | 5.45 (2.96, 8.34) | (0.80,28.60) |  | 5.05(5.10) | 3.29 (1.68, 7.07) | (0.65,10.57) |
| BDE 99 | 37.16(41.90) | 20.38 (12.90,39.40) | (3.01,133.36) |  | 55.79(57.78) | 40.91 (22.14, 60.44) | (7.29,217.99) |  | 34.77(31.45) | 27.22 (8.95, 49.20) | (4.80,72.18) |
| BDE 100 | 29.82(27.35) | 20.86 (11.14,39.78) | (2.84,90.77) |  | 42.80(39.65) | 29.76 (21.16, 50.09) | (3.55,142.54) |  | 26.21(21.56) | 18.55 (11.36, 30.05) | (7.05,67.11) |
| BDE 153 | 25.62(23.34) | 17.55 (8.60,34.20) | (2.67,78.09) |  | 41.77(34.57) | 29.42 (16.92, 68.21) | (6.59,125.07) |  | 27.73(20.45) | 20.47 (15.27, 34.83) | (5.51,71.37) |
| BDE 183 | 1.49(5.11) | 0.63 (0.45,0.84) | (0.28,2.11) |  | 0.72(0.57) | 0.56 (0.46, 0.73) | (0.30,1.64) |  | 0.69(0.26) | 0.76 (0.50, 0.85) | (0.26,1.01) |
| BDE 197 | 2.21(3.17) | 1.26 (0.71,2.60) | (0.33,6.38) |  | 1.46(1.15) | 1.26 (0.54, 1.94) | (0.31,4.35) |  | 1.93(2.02) | 1.35 (0.95, 2.20) | (0.66,3.34) |
| BDE 207 | 2.66(1.78) | 2.15 (1.71,2.80) | (1.15,6.99) |  | 2.04(1.04) | 1.90 (1.58, 2.22) | (0.27,3.39) |  | 3.20 (4.03) | 2.12 (1.37, 2.67) | (1.17,7.20) |
| BDE 209 | 4.44(6.87) | 2.96 (1.88,4.84) | (0.80,9.80) |  | 3.47(2.59) | 2.50 (1.39, 5.38) | (0.61,8.25) |  | 5.39 (7.61) | 2.40 (1.84, 3.65) | (1.01,21.41) |

*For some PBDEs, the rank ordering of the three groups based on the means differs from that based on the medians due to differences in the tails of the distributions.

Supplemental Table 1c: Comparison of mean BDEs (*pmol/g lipid*) across three groups*

|  | **AU/ASD (n=49)** | | |  | **DD (n=24)** | | |  | **TD (n=21)** | | |
| --- | --- | --- | --- | --- | --- | --- | --- | --- | --- | --- | --- |
| **PBDE congener** | **Mean(SD)** | **Med (25^th^,75^th^)** | **(5^th^,95^th^)** |  | **Mean(SD)** | **Med (25^th^,75^th^)** | **(5^th^,95^th^)** |  | **Mean(SD)** | **Med (25^th^,75^th^)** | **(5^th^,95^th^)** |
| BDE 28 | 3.50(2.66) | 2.73 (1.51,5.23) | (0.62,8.24) |  | 4.31(3.28) | 3.38 (2.32,4.85) | (0.90,11.95) |  | 3.40(2.83) | 2.16 (1.44,4.59) | (0.74,6.56) |
| BDE 47 | 175.36(146.38) | 130.50 (70.72,241.21) | (19.97,458.99) |  | 233.48(216.56) | 169.19 (112.42,246.38) | (17.72,788.25) |  | 151.05(101.31) | 151.55 (61.90,202.06) | (30.63,340.15) |
| BDE 66 | 1.33(1.36) | 0.93 (0.46,1.78) | (0.19,5.03) |  | 1.93(1.73) | 1.44 (0.80,2.06) | (0.33,6.32) |  | 1.37(1.09) | 1.27 (0.42,1.65) | (0.26,3.06) |
| BDE 85 | 7.62(7.85) | 4.61 (2.72,8.40) | (0.68,22.58) |  | 13.46(14.29) | 9.64 (5.25,14.76) | (1.42,50.64) |  | 8.95(9.03) | 5.83 (2.98,12.52) | (1.15,18.71) |
| BDE 99 | 65.80(74.18) | 36.08 (22.84,69.76) | (5.33,236.12) |  | 98.77(102.29) | 72.42 (39.21,107.10) | (12.90,385.96) |  | 61.56(55.68) | 48.20 (15.85,87.10) | (8.5,127.80) |
| BDE 100 | 52.80(48.42) | 36.93 (19.73,70.44) | (5.03,160.71) |  | 75.78(70.20) | 52.69 (37.47,88.68) | (6.29,252.38) |  | 46.41(38.18) | 32.85 (20.11,53.20) | (12.49,118.83) |
| BDE 153 | 39.80(36.25) | 27.26 (13.36,53.13) | (4.15,121.31) |  | 64.89(53.70) | 45.70 (26.28,105.97) | (10.24,194.30) |  | 43.08(31.77) | 31.80 (23.73,54.10) | (8.56,110.88) |
| BDE 183 | 2.06(7.06) | 0.88 (0.63,1.16) | (0.39,2.93) |  | 0.99(0.79) | 0.78 (0.64,1.00) | (0.41,2.27) |  | 0.95(0.36) | 1.05 (0.70,1.17) | (0.36,1.40) |
| Σ BDE 28 - 153 | 346.21(293.89) | 233.31 (137.73,441.25) | (39.10,924.64) |  | 492.62(446.41) | 351.02 (234.24,542.27) | (74.42,1635.46) |  | 315.81(223.04) | 288.28 (146.09,376.59) | (74.38,723.31) |
| Σ BDE 28 - 183 | 348.27(293.69) | 234.09 (145.10,442.29) | (39.92,925.32) |  | 493.62(446.99) | 351.78 (235.10,542.89) | (75.33,1636.57) |  | 316.76(223.17) | 289.29 (147.17,377.64) | (75.61,724.55) |
| BDE 197 | 2.76(3.96) | 1.58 (0.88,3.24) | (0.41,7.97) |  | 1.82(1.43) | 1.57 (0.68,2.42) | (0.38,5.43) |  | 2.41(2.52) | 1.69 (1.19,2.75) | (0.83,4.17) |
| BDE 207 | 3.03(2.02) | 2.44 (1.94,3.19) | (1.31,7.94) |  | 2.31(1.18) | 2.16 (1.79,2.53) | (0.30,3.85) |  | 3.64(4.58) | 2.40 (1.56,3.04) | (1.33,8.18) |
| BDE 209 | 4.63(7.16) | 3.08 (1.96,5.04) | (0.83,10.22) |  | 3.62(2.70) | 2.61 (1.45,5.61) | (0.64,8.60) |  | 5.62(7.93) | 2.50 (1.92,3.81) | (1.05,22.32) |
| Σ BDE 197-209 | 10.41(10.36) | 7.16 (5.24,11.76) | (3.19,26.94) |  | 7.75(4.73) | 6.85 (4.15,9.90) | (2.12,15.87) |  | 11.66(14.53) | 6.07 (5.12,8.42) | (4.48,33.25) |
| Σ BDE 28 - 209 | 358.68(294.85) | 290.08 (149.73,455.09) | (44.52,928.51) |  | 501.37(449.44) | 360.94 (239.98,550.31) | (80.81,1641.56) |  | 328.43(227.35) | 295.37 (152.43,391.80) | (80.34,736.05) |

*For some PBDEs, the rank ordering of the three groups based on the means differs from that based on the medians due to differences in the tails of the distributions.
